# Safety and Efficacy of Latitud™ Hip Replacement in Total Hip Arthroplasty: An Observational Study in Kazakhstan

**DOI:** 10.7759/cureus.82911

**Published:** 2025-04-24

**Authors:** Olzhas Bekarissov, Arman Batpen, Aleksey Belokobylov, Timur Baidalin, Durdana Oktyabrova, Askarjan Beknazarov, Konstantin Petrovskiy, Kiran Kumar Shetty

**Affiliations:** 1 Traumatology and Orthopedics, National Scientific Center of Traumatology and Orthopedics named after academician N.D. Batpenov, Astana, KAZ; 2 Product Performance and Engineering, Meril Life Sciences, Vapi, IND

**Keywords:** harris hip score, osteoarthritis, range of motion, surgery, total hip replacement

## Abstract

Objectives: Total hip replacement (THR) is one of the most successful surgical procedures globally for managing end-stage hip osteoarthritis. Latitud™ (Meril Healthcare Pvt. Ltd., Gujarat, India) hip replacement system is at the forefront of restoring mobility and improving patient outcomes. This study aimed to evaluate the safety and effectiveness of the Latitud™ hip replacement in total hip arthroplasty.

Methods: In this retrospective, single-center, post-market, observational study, patients who underwent THR and were treated in our hospital from 2018 to 2020 were included. The primary outcome was the THR-related intra- and postoperative complications and revision rate at one-year follow-up. Secondary outcomes were other patient-reported outcomes, radiographic analysis, Harris hip score, adverse events, implant dislocation, and implant survivorship rate. Data were collected using a pre-designed data collection form.

Results: A total of 150 patients with a mean age of 53.90 ± 11.44 years were enrolled. The surgical procedures were successful without any major complications. There was a significant (p < 0.001) improvement in the Harris hip score. No death was observed throughout the study. There was one (0.67%) intra-operative fracture, with the patient having a closed comminuted fracture of the upper and middle third of the left femur and anemia. Seven patients (4.67%) required postoperative blood transfusions. During the follow-up period, there was no revision surgery or implant dislocation.

Conclusion: This observational study suggests that the Latitud™ hip replacement system was favorable in terms of safety and effective in a real-world setting in Kazakhstan. The study showed promising results in pain alleviation, functional improvement, and implant survivorship.

## Introduction

Hip fractures are serious medical conditions associated with impaired mobility, reduced autonomy, and increased morbidity and mortality. These fractures typically occur either intracapsularly (e.g., femoral neck fractures) or extracapsularly (e.g., intertrochanteric and subtrochanteric fractures) [1]. Total hip replacement (THR) is an effective joint replacement procedure used to alleviate pain and improve mobility in patients with severe osteoarthritis (OA) or rheumatoid arthritis [2,3]. It is also regarded as a successful surgical intervention that can even restore athletic function, supported by surgeon-reported outcomes and gait analysis [4]. However, patient-reported outcome measures indicate that some patients continue to experience pain and functional limitations post-THR [5]. Identifying factors such as preoperative pain sensitization is crucial to improving patient outcomes through targeted interventions.

Hip prostheses replace damaged hip joints, which are anatomically composed of the femoral head inserted into the acetabulum, protected by articular cartilage within a synovial capsule. Hip contact forces are substantial, peaking at approximately 260% of body weight during stair descent [6]. The greatest torsional moment with a prosthesis occurs during unassisted walking and stair ascent [7]. These forces, coupled with predisposition to OA, contribute to pain.

Modern prosthetic systems are derived from Charnley’s 1960s low-friction arthroplasty design and typically consist of a metal femoral stem and ball with an acetabular component made of crosslinked polyethylene [8]. Femoral stems and acetabular components can be cemented or press-fitted, with optional screws for stabilization. Prosthetic complications include dislocation, component loosening, and wear, influenced by patient, surgical, and prosthetic factors. Metal-on-metal bearings have been associated with adverse reactions like osteolysis and metallosis, whereas ceramic-on-ceramic bearings reduce fracture risk but may cause squeaking. Evaluating wear, corrosion, and femoral component fatigue is essential in assessing prosthesis performance. This study evaluates the safety and performance of the Latitud™ hip replacement system (Meril Healthcare Pvt. Ltd., Gujarat, India) used for THR.

This manuscript was previously made available as a preprint on the Research Square preprint server on July 30, 2024.

## Materials and methods

Ethical approval of the study

The study has been approved by the Ethics Committee for human subject participation (Vide. Commission Meetings on Ethics and Ethical Evaluation of Clinical Trials in Republican State Enterprise (RSE) on Republican Expert Meeting (REM), May 12, 2021; No. 07-02-05-08/0873U). As this retrospective analysis did not involve changes to patient management, the Ethics Committee provided a waiver for written informed consent. The study adhered to ICH-Good Clinical Practice (GCP), ISO 14155:2020, and the Declaration of Helsinki ethical standards [9].

Study design

This retrospective, single-center, post-market study included 150 patients who underwent THR using the Latitud™ hip replacement system between July 2018 and August 2020 at the National Scientific Center of Traumatology and Orthopedics, Kazakhstan. Patients with significant osteoporosis, compromised bone stock, or metabolic disorders associated with systemic bone degeneration were excluded. The primary objective was to evaluate the safety and clinical performance of the Latitud™ hip replacement system under real-world conditions.

Data collection and patient demographics

Patient identities were anonymised, and data collection was conducted in accordance with applicable regulatory guidelines. Baseline characteristics, demographic information, procedural details, discharge summaries, and data on both primary and secondary outcomes were extracted from medical records and systematically documented using case report forms. All reported adverse events and serious adverse events occurring intraoperatively or postoperatively were recorded. Additionally, any history of substance addiction was documented, if present.

Components of the Latitud™ hip replacement system

**Figure 1:**
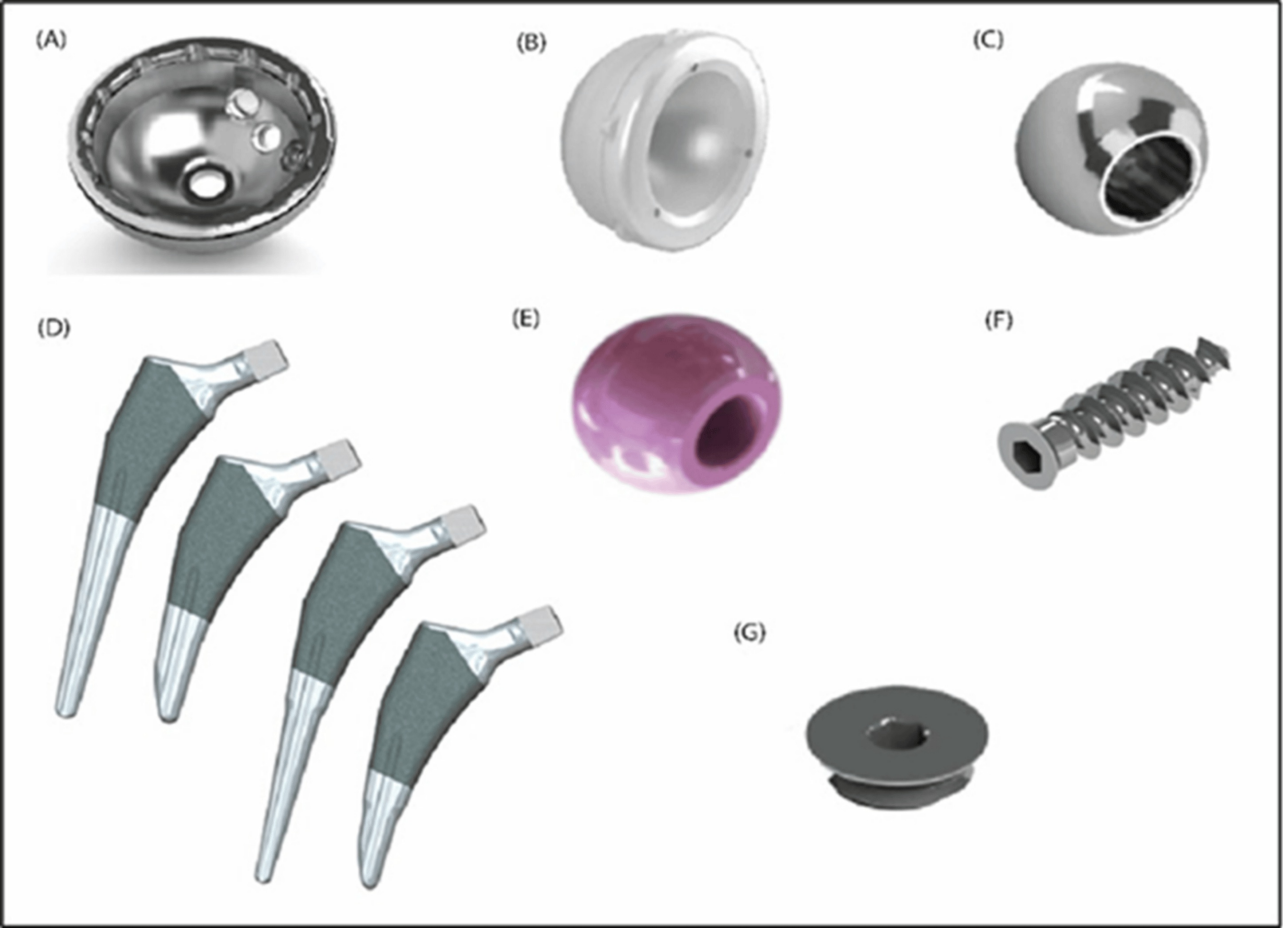
Components of Latitud™ hip replacement system: (A) modular shell (viz. acetabular cup); (B) modular liner (viz. acetabular liner); (C, E) modular femoral head; (D) uncemented/cemented femoral stem; (F) bone screw; (G) apical hole occluder. All images and illustrations presented in this manuscript were created by Meril Life Sciences and are proprietary to Meril Healthcare Pvt. Ltd. Authors confirm that permission to reproduce the image has been obtained from Meril Healthcare Pvt. Ltd.

The Latitud™ hip prosthesis system comprises an acetabular cup, outer polyethylene acetabular liner, locking ring, bipolar ring, prosthetic head, and femoral stem (cemented or uncemented) (Figures 1A-1G).

The acetabular cup, made from titanium alloy ELI (Ti6Al4V, ASTM F136), is intended for cementless fixation and coated with pure titanium for enhanced fixation. The liner is made from highly crosslinked polyethylene (HXLPE, ASTM F648), and the femoral head from cobalt-chromium alloy (ASTM F1537-1).

Femoral stems come in uncemented and cemented variants. The uncemented stem, made from titanium alloy ELI (ASTM F136), is hydroxyapatite-coated for better fixation and features a 12/14 taper to pair with the modular head, used without bone cement. The cemented stem, made from high-nitrogen stainless steel (ISO 5832-9:2019), is cement-fixed and also has a 12/14 taper. The bipolar shell sizes range from 37 mm to 63 mm, and the femoral head is 22 mm or 28 mm.

Surgical procedure

The surgical team used the “Transgluteal” approach, also known as the Direct Lateral Approach (DLA), modernized by Hardinge in 1982 [10,11]. Patients were positioned in either the supine or lateral decubitus position, per the surgeon's preference. During intraoperative imaging, they were placed on a standard or radiolucent table, with a bump under the anterior superior iliac spine (ASIS) for femoral displacement and a roller bar under the ipsilateral calf for leg stabilization.

The incision began proximal to the anterior-middle third of the greater trochanter (GT) and extended distally along the femur. The fascia lata and iliotibial band (ITB) were incised, and the gluteus medius was split using blunt dissection. The anterior structures were elevated subperiosteally, and femoral neck osteotomy was performed after hip dislocation.

Acetabular exposure was obtained using three retractors, followed by routine acetabular preparation. The leg was placed in a figure-four position for femoral preparation, and retractors lateralized the proximal femur to visualize the shaft and version. After component placement, the anterior flap was repaired, and closure of the fascia lata, ITB, gluteus maximus, subcutaneous tissues, and skin followed.

Outcomes

The primary outcome was a composite measure comprising intraoperative complications, postoperative complications, and revision rates. Intraoperative complications included events such as fractures and neurovascular injuries occurring during surgery. Postoperative complications encompassed aseptic loosening, infection, and persistent pain. Revision rate referred to the proportion of patients requiring component removal or replacement. Secondary outcomes included implant dislocation, prosthesis survivorship, radiographic assessments, Harris Hip Score (HHS), mobility status, length of hospital stay, and incidence of adverse events. Together, these outcomes provided a comprehensive evaluation of the safety and clinical success of the Latitud™ hip replacement system.

Clinical follow-up and assessment

At the time of reporting, patients had a follow-up period of at least 1.5 to two years. Routine follow-ups after THR included x-ray imaging and clinical examinations to assess the active range of motion (AROM) of the hip joint [12,13]. AROM was measured using a goniometer at baseline and post-procedure to evaluate changes in joint movement [14].

For abduction/adduction, patients were positioned supine, and the goniometer was aligned with the anterior midlines of the pelvis and femur along the x-axis. Rotational angles (y-axis) were measured with patients sitting or prone, hips and knees flexed at 90˚, recording internal/external rotation. Flexion/extension (z-axis) was assessed with the patient supine, aligning the goniometer with the lateral midlines of the pelvis and femur.

Gait pattern assessments were also conducted during clinical examinations to evaluate hip function under load. The HHS, on a 0-100 scale, assessed pain, daily activities, and hip function by evaluating range of motion (ROM) before and after implantation [15-17].

Statistical analysis

Microsoft Excel and SPSS version 27.0 (IBM Corp., Armonk, NY) were used for data cleaning and statistical analysis. Kolmogorov-Smirnov was applied to assess the data normality. Categorical and continuous variables were reported in proportions and mean ±SD. Paired Sample T Test was used to assess the change in mean between two dependent groups. Venny 2.1 (Centro de Investigación Príncipe Felipe (CIPF), Valencia, Spain) was used to build the Venn diagram [18]. The statistical significance was determined at a 5% level of significance.

## Results

Clinical and demographic parameters of the patients

This study included 150 patients of various ethnicities and age groups (Table 1). The cohort comprised 74 (49.3%) males and 76 (50.7%) females, both with higher BMI values. At admission, 11 patients (7.33%) reported alcoholism, and nine (6%) were smokers. Eleven patients had less than 10˚ fixed internal rotation, while 12 had less than 30˚ fixed flexion contracture, and 12 patients had restricted internal rotation in extension to 10˚. An abduction angle below 10˚ was noted in eight patients. Common co-morbidities included lumbar osteochondrosis, sciatica, dysplastic coxarthrosis, post-traumatic aseptic necrosis, ankylosing spondylitis, and rheumatoid polyarthritis, contributing to joint degeneration and the need for surgery. A total of 90 (60%) patients underwent right-side hip replacement, 52 (34.7%) had left-side replacements, and eight (5.3%) had both hips replaced.

**Table 1:** Demographics of patients

Variables	Frequency (%) (n, 150)
Age (mean)	
Male	52.6 ± 11.0
Female	55.2 ± 11.8
Gender	
Male	74 (49.3)
Female	76 (50.7)
Ethnicity	
Kazakh	83 (55.3)
Russian	66 (44)
Ukrainian	1 (0.7)
Race	
Asian	82 (54.7)
European	68 (45.3)
BMI (mean)	
Male	26.49 ± 4.15
Female	27.31 ± 5.09
Affected hip joint	
Right	90 (60)
Left	52 (34.7)
Both	8 (5.3)
Medical history	
Cardiovascular	35 (23.3)
Arterial hypertension	3 (2.0)
Ischemic heart disease	1 (0.7)
Cardiac ischemia	5 (3.3)
Angina pectoris	1 (0.7)
Atherosclerotic cardiosclerosis	3 (2.0)
Congestive heart failure	1 (0.7)
Normosystolic constant form	35 (23.3)
Gastrointestinal & hepatic	
Gastritis	6 (4.0)
Chronic catarrhal gastroduodenitis	1 (0.7)
Gastroesophageal reflux disease (GERD)	1 (0.7)
Cholelithiasis	2 (1.3)
Chronic cholecystitis	3 (2.0)
Chronic pancreatitis	2 (1.3)
Chronic viral hepatitis C	1 (0.7)
Metabolic & endocrine	
Diabetes mellitus type 2	5 (3.3)
Hypothyroidism	6 (4.0)
Obesity	14 (9.3)
Respiratory	
Acute respiratory disease	1 (0.7)
Acute sinusitis	1 (0.7)
Neurological & musculoskeletal	
Spinal osteochondrosis	3 (2.0)
Sciatica (left)	1 (0.7)
Ankylosing spondylitis (axial form)	1 (0.7)
Bechterew’s disease	2 (1.3)
Bone ankylosis	2 (1.3)
Ossification of longitudinal intervertebral ligament	1 (0.7)
Rheumatoid polyarthritis	1 (0.7)
Hereditary sensory neuropathy	2 (1.3)
Hematologic	
Anemia	2 (1.3)
Urogenital	
Chronic pyelonephritis	1 (0.7)
Other	
Ventral hernia	1 (0.7)
Right-sided direct inguinal hernia	1 (0.7)
Telangiectasia of lower extremities	2 (1.3)
Allergic reaction	3 (2.0)
Substance dependency	
Alcoholism	11 (7.33)
Tobacco smoking	9 (6)

Procedural outcomes of the THR

**Figure 2:**
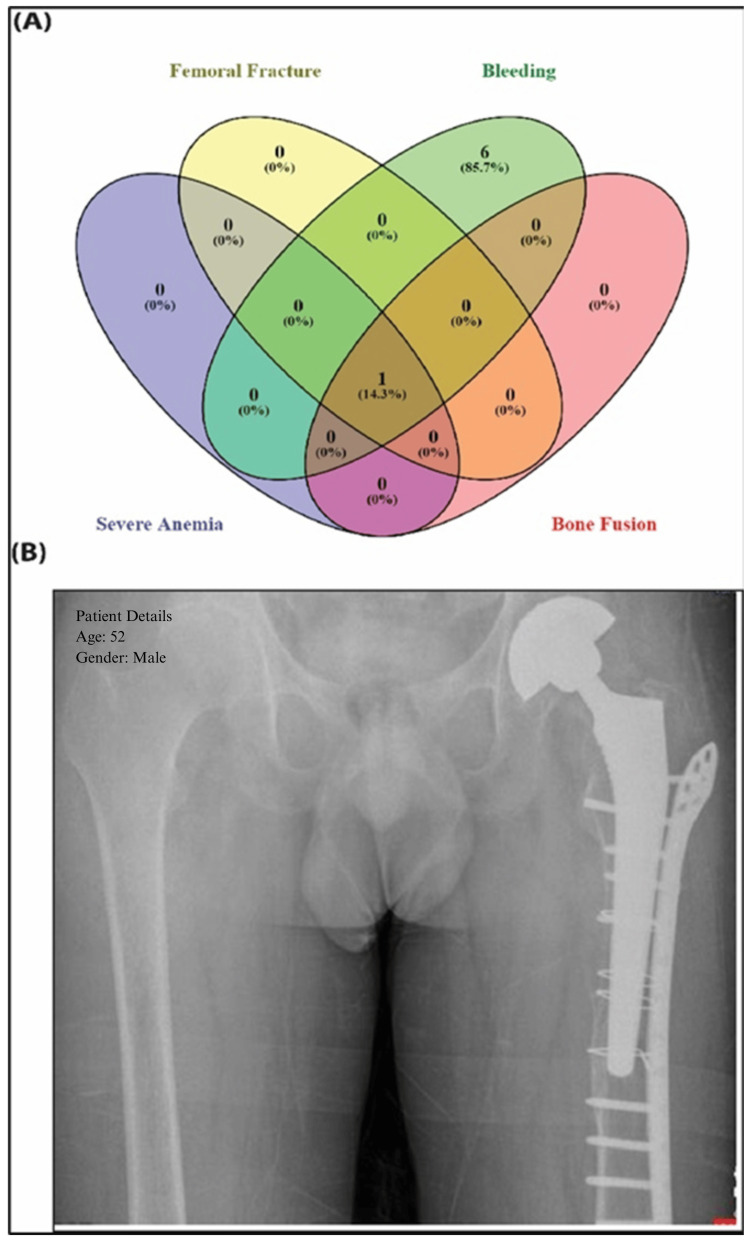
(A) The Venn diagram encompassed all the intra- and postoperative adverse events and complications. The peri-operative complications were mainly pain (n=150) and bleeding (n=7). A femoral fracture happened intra-operatively in one patient, and it had a bone fusion at the site of fracture later. (B) The radiograph image of a patient showed femoral fracture and a hip fracture osteosynthesis with a plate and wire cerclages. The patient had chronic osteoporosis and also suffered from severe bleeding postoperatively.

All 150 patients (100%) successfully underwent surgery without intraoperative mortality and postoperative period (two years). The mean surgery duration was 56.5 ± 14.2 min, and hospital stays averaged 12.2 ± 2.4 days. No neurovascular injuries or major adverse events occurred during the Latitud™ System implantation, and no technical difficulties were reported. Minor postoperative complications were observed, with pain being the most common and eventually subsiding. Seven patients (5%) experienced bleeding, with seven (5%) requiring blood transfusions. One patient (0.7%) developed severe anemia related to the procedure and suffered a periprosthetic fracture, necessitating additional surgical repair with a locking plate and cerclage. Bone fusion was achieved, as demonstrated in Figures 2A, 2B, without any postoperative complications or need for further treatment.

Postoperative modulations of hip joint-related symptoms

At discharge, most patients had improved symptoms without additional health concerns. Pre-operatively, patients had varying limp scores (0-11; mean 5.92 ± 1.76), with 35% having no/slight limp (scores 8 or 11). Postoperatively, all patients were able to walk without a limp, with a mean score of 10.13 ± 1.41. Prior to surgery, external support was commonly required (score range: 3-11; mean 6.22 ± 1.28). Following THR, 95% of patients required no support or used only a cane for extended ambulation, with a mean score of 9.74 ± 1.98. Walking distances improved significantly, with 72% walking six blocks or more post-surgery (score 11) compared to limited distances pre-surgery (mean 6.40 ± 1.74). Difficulty with socks/shoes improved from preoperative scores of 0-4 (mean 1.88 ± 0.81) to 2-4 (mean 3.88 ± 0.47). Sitting comfort increased from 0-4 (mean 3.00 ± 0.66) to 2-5 (mean 4.24 ± 0.68). Public transport use showed minimal change post-THR (mean 1.33 ± 0.47) versus preoperative scores (1.62 ± 0.52). Stair ascent scores improved from 0-4 (mean 1.92 ± 0.35) to 4 (mean 3.87 ± 0.50).

**Figure 3:**
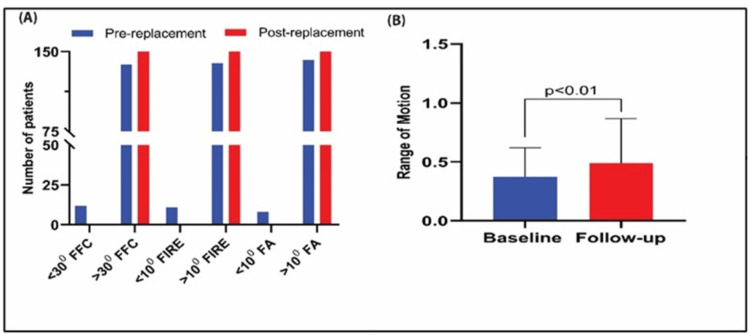
(A) Limitation of any particular range of motion is denoted as the fixed angle of movement. All measured fixed angles of movement, namely fixed flexion contracture (FFC), fixed internal rotation in extension (FIRE), and fixed abduction (FA) showed significant (p-value <0.05; Paired t-test) improvement post-THR. All the patients (n=150) were able to augment their range >30˚ for FFC and >10˚ for FIRE and FA. (B) We observed that there was a cumulative augmentation in ROM value by 1.31-fold (p<0.01) due to the successful implantation of Latitud™. THR: Total hip replacement

Twelve (8%) patients had <30˚ fixed flexion contracture pre-THR, which resolved postoperatively. Internal rotation was <10˚ in 11 (7.3%) patients pre-surgery; all exceeded 10˚ after. Eight (5.3%) patients had preoperative abduction <10˚, which improved post-surgery (Figure 3A). Three (2%) patients with limb length discrepancies <3.2 cm were corrected. Overall ROM improved significantly from 0.37 ± 0.25 to 0.49 ± 0.37 (p<0.01), with increases in 64 (42.6%) patients, decreases in 48 (32%), and no change in 38 (25.4%) patients (Figure 3B).

Flexion improved from 70°-110° pre-surgery to 80°-110° post-surgery in 98% of patients. Abduction improved from 5˚-20° pre-surgery to 15°-20° in 91% post-surgery. External rotation improved from 5°-15° to 10°-15° in 93% of patients. Adduction improved from 5°-20° to 10°-20° in most cases (Table 2).

**Table 2:** Comparison of alterations in different angles of movement

Degree of movement	Pre-replacement				Post-replacement			
	Total flexion (^0^) (n=146)	Total abduction (^0^) (n=146)	Total external rotation (^0^) (n=146)	Total adduction (^0^) (n=146)	Total flexion (^0^) (n=150)	Total abduction (^0^) (n=150)	Total external rotation (^0^) (n=150)	Total adduction (^0^) (n=150)
5˚-10˚ (%)	—	8 (5.48)	48 (32.88)	31 (21.23)	—	—	11 (7.33)	11 (7.33)
10˚-15˚ (%)	—	74 (50.68)	98 (67.12)	115 (78.77)	—	13 (8.67)	139 (92.67)	139 (92.67)
15˚-20˚ (%)	—	64 (43.84)	—	—	—	137 (91.33)	—	—
70˚-75˚ (%)	2 (1.37)	—	—	—	—	—	—	—
75˚- 80˚ (%)	11 (7.53)	—	—	—	3	—	—	—
80˚-90˚ (%)	61 (41.78)	—	—	—	10	—	—	—
90˚-100˚ (%)	65 (44.52)	—	—	—	56	—	—	—
100˚-110˚ (%)	7 (4.79)	—	—	—	81	—	—	—

**Figure 4:**
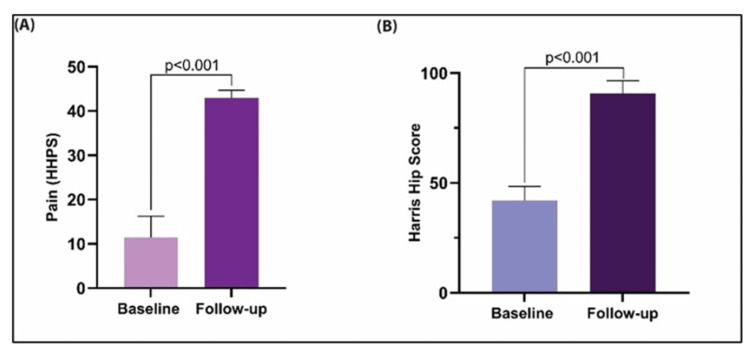
(A) HHS-based pain score or HHPS is inversely proportional to the pain intensity while performing activities that involve hip joint mobility. The HHPS was enhanced by 3.75-fold at least one year after the implantation procedure (p<0.01; Paired t-test). (B) The HHS improved significantly by 2.2-fold post-THR (p<0.001; Paired t-test), indicative of better functional range of the patients bearing the Latitud™ prosthesis. HHS: Harris hip score, HHPS: Harris Hip Pain Score, THR: Total hip replacement

Postoperative pain scores, as measured by the Harris Hip Pain Score (HHPS), significantly improved from a baseline mean of 11.45 ± 4.84 to 42.93 ± 1.77 (p < 0.001), reflecting a marked reduction in pain. Similarly, the mean HHS increased from 42.3 ± 7.1 preoperatively to 91.1 ± 5.2 at one-year post-surgery (p < 0.001), indicating a substantial improvement in clinical outcomes (Figures 4A, 4B).

## Discussion

With the growing burden of bone degeneration-related diseases, total joint arthroplasty (TJA) is among the most common orthopedic procedures [19,20], with total hip arthroplasty (THA) on the rise due to the prevalence of osteoarthritis (OA) in 10%-15% of adults over 60 [21].

This study evaluated the safety and efficacy of the Latitud™ hip replacement system in 150 patients from Kazakhstan over a follow-up of at least one year. The results indicated that the Latitud™ system is a viable option for THR, providing significant pain relief, functional improvements, and implant survival. Patients underwent unilateral or bilateral THR using the DLA method, known for accurate component positioning and efficient hip biomechanics restoration. Despite co-morbidities such as rheumatoid polyarthritis and osteochondrosis and risk factors like alcoholism and smoking, the system demonstrated a strong safety profile. Only one intraoperative fracture, due to pre-existing osteoporosis, required conservative treatment and resulted in bone fusion. Postoperative bleeding occurred in 5% of patients but was managed with transfusion. No neurovascular injuries were noted, and only one patient developed severe anemia needing transfusion, supporting the system’s safety.

Patients experienced pain relief and improved mobility, with most walking without a limp, climbing stairs without support (94%), walking moderate-to-long distances with minimal assistance (95%), and easily wearing shoes/socks (96%). THR did not significantly impact public transport use. These factors enhanced patients’ quality of life, showcasing the Latitud™ system’s superior performance and low adverse event incidence. The one patient with an intraoperative fracture had ankylosing spondylitis, osteoporosis, and moderate anemia, with no evidence linking hormone dependence or perioperative complications.

HHS, a widely used health status scale for more than 50 years [22,23], demonstrated significant ROM improvements in flexion, extension, abduction, and rotation post-implantation, though 48 patients showed reduced ROM and 38 showed no change. Overall, improved HHS indicated cumulative functional gains. Limb length discrepancies [24] were corrected in three patients, and fixed-angle limitations were resolved post-surgery. Surgery duration and hospital stay were comparable to previous studies, affirming the system's efficiency [25,26]. The device also improved the symptoms associated with degenerative hip joints, as established by the enhanced HHS index.

A Global Burden of Disease study in Kazakhstan involving 56,895 OA patients found women were three times more likely to develop gonarthrosis [27]. This study tested the Latitud™ system in a population heavily impacted by degenerative bone disease. This mid-term study highlights the system's potential for long-term benefits, implant survival, and low complication rates. Extended follow-ups and multicenter studies are recommended to confirm these findings across broader populations.

Study strength

To our knowledge, this is among the first post-market evaluations of the Latitud™ Hip Replacement System conducted in Kazakhstan, offering valuable insights into its safety and performance in a real-world clinical setting. The use of a standardized surgical approach and consistent postoperative follow-up strengthened the internal validity of the findings. Additionally, comprehensive clinical, radiographic, and patient-reported outcomes (including HHS and ROM metrics) were collected, offering a holistic view of patient recovery and implant performance.

Study limitation

The retrospective and single-center design may limit the generalizability of the findings to other populations and healthcare settings. Additionally, the follow-up period of approximately 1.5 to two years, while adequate for mid-term outcomes, may not capture long-term complications or prosthesis longevity. The relatively small sample size of 150 patients and data collection based on medical records and patient-reported outcomes may introduce recall bias and inaccuracies. Furthermore, the absence of a control group restricts the ability to directly compare outcomes with other hip replacement systems or surgical approaches.

Future direction

Given the growing burden of musculoskeletal disorders in Central Asia and the increasing demand for joint arthroplasty in Kazakhstan, future studies should adopt a multicenter, prospective design with longer follow-up durations. Comparative analyses between different implant systems and surgical approaches (e.g., anterior vs. lateral) would be valuable. Integrating health-economic evaluations and patient satisfaction metrics would also provide a more comprehensive understanding of the intervention’s impact. Moreover, establishing a national joint replacement registry in Kazakhstan could facilitate longitudinal tracking of outcomes and complications, enhancing national data quality and guiding evidence-based policy decisions.

## Conclusions

Our study demonstrated that the Latitud™ hip replacement prosthesis system significantly enhances functional mobility for routine activities while maintaining a low risk of intra- and postoperative complications. The Latitud™ system effectively reduced pain, improved walking ability, increased ROM, and corrected deformities in patients undergoing THR. These findings underscore the Latitud™ system as a safe, reliable, and effective solution for total hip arthroplasty, showing sustained benefits and promising outcomes in this mid-term follow-up.
